# Removal of Environmental Nanoparticles Increases Protein Synthesis and Energy Production in Healthy Humans

**DOI:** 10.3389/fbioe.2022.800011

**Published:** 2022-02-14

**Authors:** Eduardo Antuña, Juan Carlos Bermejo-Millo, Enrique Caso-Onzain, Enrique Caso-Peláez, Yaiza Potes, Ana Coto-Montes

**Affiliations:** ^1^ Department of Morphology and Cell Biology, Faculty of Medicine, University of Oviedo, Oviedo, Spain; ^2^ Instituto de Investigación Sanitaria Del Principado de Asturias (ISPA), Av. Del Hospital Universitario, Oviedo, Spain; ^3^ Instituto de Neurociencias del Principado de Asturias (INEUROPA), University of Oviedo, Oviedo, Spain; ^4^ Innovación Unit, BiowAir Total Systems SL, C/Michel Faraday, Gijón, Spain; ^5^ Scientific CEO MyOmics SL, Gijón, Spain; ^6^ System and Precision Medicine, Hospital Covadonga Gijón, Gijón, Spain; ^7^ Biomedical Unit, BiowAir Total Systems SL, Gijón, Spain

**Keywords:** nanoparticles, air pollution, filtration, ATP, protein synthesis p70S6K, total antioxidant activity, mitochondria

## Abstract

Currently, industrial activity causes the environmental release of nanoparticles that have multiple adverse effects on population health. There is a clear correlation between the increase in particulate air pollution and the increases in mortality and morbidity rates in both adults and children, which demonstrates the toxic effects of these particles. However, the effect of particle removal on healthy individuals is unknown. Thus, in this preliminary study, we showed, for the first time, how the filtering equipment that we used significantly reduced a large amount of nanoparticles in a minimum time and induced a reduction of oxidative damage in healthy individuals of both sexes after 25, 50 and 100 days of exposure. These effects led to increased protein synthesis and enhanced mitochondrial efficiency, thus resulting in a highly significant triggering of ATP synthesis. These results not only provide insight into the chronic effects that environmental nanoparticles have on individuals prior to the development of pathologies but also demonstrate a system capable of reversing nanoparticle toxicity and allowing cellular energy recovery.

## 1 Introduction

Environmental pollution is an unfavorable modification of our surroundings as a result of urbanism, population growth, solar radiation, and primarily, the industrial activity that human beings have expanded in recent years. Over the last few centuries, this activity has acquired special importance because of its exponential increase and the need of human beings to identify systems that can stop the resulting pollution in view of the increasing evidence of its effects in the medium and long-term exposure.

Exposure to particulate matter is frequently associated with pathogenic processes and even mortality (Brook et al., 2010), and particle size is one of the main defining characteristics the contributes to leathality. Moreover, particles are divided according to their aerodynamic sizes. Thus, particles with an aerodynamic size range between 10 and 2.5 μm (PM10-2.5) are known as the coarse fraction, whereas particles smaller than 2.5 μm (PM2.5) are known as the fine fraction. The so-called ultrafine particles are those particles equal to or smaller than 0.1 μm, and those particles below 100 nm constitute nanoparticles (Gwinn and Vallyathan, 2006; Nemmar et al., 2013).

The proportion of nanoparticles with which we usually live, as well as the proportion that is spread in the air or contained in atomizers, has a significant influence on our state of health, due to the fact that they can be absorbed into our body through skin pores, weakened tissues and injections, as well as through olfactory, respiratory and intestinal tracts (Yah et al., 2012). Subsequently, they can be systematically disseminated, resulting in effects that have not yet been fully identified but are often harmful since they can function as cofactors or aggravators of undesirable biological events of diverse types, ranging from lung inflammation (Lopes et al., 2009) to chronic obstructive pulmonary disease (Rennard, 1998) and including different types of tumors (Nemmar et al., 2002; Warheit et al., 2004). Additionally, these effects can also result in damage to the brain (Oberdörster et al., 2004). Although these alterations have been well documented, with an increasing number of conditions being detected every day, the cellular mechanisms that are affected by the daily infiltration of nanoparticles remain practically unknown, which is a relatively recent field of health research.

The removal of these environmental nanoparticles, which is mainly accomplished by filtering processes, should significantly reduce the aforementioned effects, following a dose-response curve and depending on the sizes of the filtered particles, as well as being directly related to the basal state of the individual. However, the usual air filtering systems do not commonly interfere with the concentrations of the dispersed nanoparticles, as these particles are smaller than the pore or filtering system. This phenomenon has been widely documented in relation to the global infection of SARS-CoV-2, as the virion size is between 60 and 140 nm (Liu et al., 2020). Furthermore, air conditioners do not necessarily filter the air but can create an air recirculation system without external intake (Li et al., 2021), which keeps the concentration of nanoparticles unchanged. Therefore, effective strategies to reduce indoor air pollutant concentration events are critically needed.

Evidence shows differences between men and women in several health indicators. Globally, the life expectancy is greater for women than for men. However, women manifest higher degree of dependency in advanced ages (Institute of Medicine (US) Committee on Gender Differences in Susceptibility to Environmental Factors, 1998). Protein synthesis and cellular bioenergetics are considered as key molecular processes involved in cellular quality control and lifespan. Increased protein synthesis is associated with the improvement of cellular functioning by favoring the activation of protective pathways and the alleviation of cell death processes. Substantial alterations in protein synthesis that lead to protein malformations or misfolding eventually result in cell death (Rubio-González et al., 2020). Conversely, improvements in cellular function are evidenced by more efficient protein synthesis (Bermejo-Millo et al., 2018). The mammalian target of the rapamycin/ribosomal protein S6 kinase beta-1 pathway, which is usually known as mTOR/p70S6K, is directly involved in the control of cell metabolism and growth, and its activation is directly related to protein synthesis and degradation processes (Zanchi and Lancha, 2008). Correspondingly, a lack of energy, which is mainly induced by mitochondrial malfunction, not only slows cellular processes (Poeggeler et al., 2005), but also triggers the production of oxygen free radicals that can cause irreparable damage to cellular macromolecules (Potes et al., 2019). Proteomics is a powerful strategy that allows for a precise approach to detect the modifications that the environment causes at the cellular level (Schinzel and Dillin, 2015), which can allow us to identify the specific level at which cellular mechanisms are affected.

The characterization of the adverse effects that the inhalation of nanoparticles causes at the cellular and molecular level in the general population is significantly hampered by the difficulty of obtaining a nanoparticle-free environment without also achieving sterility, which would be toxic to controls. However, we possess air filtration equipment that allows us to achieve an environment with a significant reduction of nanoparticles. Thus, the objective of the current study was to study the effect that the combination of air filtration and plasma ionization has on human health at the systemic and cellular levels. Our obtained results showed, for the first time, how this combined strategy can induce a reduction in oxidative damage in conjunction with a substantial improvement in both protein synthesis and energy production in healthy humans.

## 2 Materials and Methods

### 2.1 Participants

Fifty-eight individuals who were randomly selected from the Asturian population constituted the BioW cohort (BioW). All of the participants were independent, healthy and consisted of individuals of both sexes (28 men and 30 women), who ranged in age from 22 to 82 years ( $\bar{x}$ = 55,59 ± 14,59). Informed consent was obtained from all subjects involved in the study.

Inclusions criteria for cohort incorporated the following: Individuals in good general health, with no relevant medical problems; well conditioned home with adequate thermal insulation; regular lifestyle with no significant alterations in daily life and adequate sleep hygiene. Barthel Index ≥80 in people over 65 years old.

Exclusion criteria included: Major illnesses and/or comorbidities; cognitive impairment; obesity (Body Mass Index BMI ≥30), irregular living, lack of adequate housing.

A longitudinal study of the temporal effect caused by the use of the nanoparticle remover (developed by BioW^©^ [BioWAir Total System SL, Spain]) constituted the BioW group. The procedure was enacted in the following manner. The system was placed in the sleeping room of the participants, in order to ensure its effect for at least 6–8 h of restful sleep. The system was kept on for 24 h a day without interruption for the entire duration of the study. Moreover, the system was programmed to maintain the level of nanoparticle removal for 24 h a day. The obtained results were compared to the baseline levels of the same individuals at time 0. Blood samples were collected prior to the use of the equipment and at 25, 50 and 100 days of use.

### 2.2 Nanoparticles Remover

During the study, the air quality in the locality was always maintained at adequate levels in terms of the concentration of suspended particulate matter, according to current Spanish legislation (Royal Decree 102/2011).

Nanoparticles were removed from the indoor space by applying an automatic, multilayer, heterogeneous broad spectrum nanofiltration system that is unique to the continuous generation of fluxes (BioW^©^). The system automatically integrates a complex continuous process, including 1) an inlet prefilter for the removal of larger impurities, 2) a bactericidal sheet, 3) a HEPA 13 filter (High Efficiency Particle Arresting), 4) an activated carbon layer that absorbs volatile substances (gases, fumes and odors) and eliminates volatile organic compounds (VOCs), 5) a heavy-duty stainless-steel filter designed for high airflows and ultraviolet (UV) baffle for platinum catalysis, 5) ultraviolet (UV) light for the denaturation of bacterial and viral genetic material, 6) a plasma ionizer to generate a charge change of the nanoparticles, thus inducing their agglutination for more effective filtering and 6) a plate for thermal sterilization at 200°C prior to laminar flow exit from the system.

The electrical installation of the enclosure where it was be used complies with EC regulations. In this study, the device was placed in the bedroom, it was kept at a distance of no more than 2 m from the bed and the automatic mode was activated, in which the screen turns off in the absence of light and turns on in the presence of brightness. Thus, although the equipment would be on 24 h a day, it is expected that the most direct and effective effect would be realized during sleeping hours, which is the reason for the exclusion of volunteers who did not maintain an adequate sleep/wakefulness hygiene.

The equipment does not require any additional measures to be adopted in conditions that can be reasonably predicted, with regards variations in pressure, acceleration or thermal ignition sources. In terms of equipment fixed costs, in the study the devise was free of charges, and no facility expenditures were necessary to prepare the site for system installation. Among variable costs, recurring cost for the operation of the system on electrical consumption (<22,4 W) was less than 1,00€ per month along the 3 months of study. Although, the system only requires filter annual replacement, due to the study period of time, there was no added cost for supply, material and maintenance during the 100 days the study protocol.

### 2.3 Nanoparticles Determination

The level of particulate matter (PM) in suspension (sizes 0.3; 0.5; 1.0; 2.5; 5.0 and 10) was tested by using a PC-220 (TROTEC) portable particle counter (Calibrated Serial number: 17020034, SGS Tecno SA & Comfort Direct) on indoor environmental samples from space dimensions that ranged between 23.75 m^3^ of minimum volume and 183.60 m^3^ of maximum volume. Tests on the volumetric flow rate, the air velocity and the outlet temperature of the system were also performed.

### 2.4 Blood Collection

Blood samples were extracted via venipuncture after an overnight fast. All of the venous blood samples were obtained before 10:00 a.m. to preclude circadian variation.

Collected blood samples were fractionated into plasma, erythrocytes and Peripheral Blood Mononuclear Cells (PBMC) in a Ficoll-Paque Plus gradient (Ficoll-Paque Plus GE Healthcare) and stored at −80°C until further analysis, as has been previously described (de Gonzalo-Calvo et al., 2010, 2012). The Bradford method (Bradford, 1976) was used to measure plasma protein concentrations.

### 2.5 Oxidative Stress Studies

Protein oxidative damage (PD) was measured in plasma by using the methods described by Levine et al. (Levine et al., 1990), with modifications of Coto-Montes and Hardeland (Coto-Montes and Hardeland, 1999), for the determination of the concentrations of carbonylated proteins. The results are presented as nmol carbonylated protein/mg protein.

Total antioxidant activity (TAA) was determined in plasma by using the ABTS/H_2_O_2_/HRP method (Arnao et al., 2001), which was modified in our laboratory for plasma samples (de Gonzalo-Calvo et al., 2010, 2012). The results are expressed as equivalents of mg Trolox/g protein.

### 2.6 Western Blot Inmunoassay

For the different western blot immunoassays, tissue homogenates (50 µg of protein per sample) were mixed with Laemmli sample buffer (BioRad Laboratories, Inc., CA, United States). The samples were fractionated by using SDS-PAGE at 200 V. After separation, protein was transferred to polyvinylidene fluoride membranes at 350 mA (Immobilon TM-P; Millipore Corp., MA, United States). β-actin (typical housekeeping protein) no showed significant variations in their levels; therefore, it was used to ensure equal loading. The membranes were incubated with the following primary antibodies: p-70S6 kinase (9,202, Cell Signaling); phospho-p-70S6 kinase (9,206, Cell Signaling); CI-20 (NDUFB8) (ab110242, Abcam, Cambridge, United Kingdom); CII-30 (SDHB) (ab14714, Abcam, Cambridge, United Kingdom) and CV (ATP5A) (ab14748, Abcam, Cambridge, United Kingdom). The membranes were incubated with the corresponding horseradish peroxidase-conjugated secondary antibody (Sigma-Aldrich, Missouri, United States). The membranes were developed using a chemiluminescent horseradish peroxidase substrate (WBKLS0500, Millipore Corp., Darmstadt, Germany), according to the manufacturer’s instructions. Image Studio Lite 5.2.5 software (LI-COR Biosciences, Nebraska, United States) was used for the quantitative analysis.

### 2.7 ATP Production

Adenosine triphosphate (ATP) levels were determined by using a commercially available ATP bioluminescent kit (FLAA, Sigma-Aldrich, Saint Louis, MO, United States). The assay was performed as indicated by the manufacturer. The results are expressed as nmol ATP/g protein.

### 2.8 Statistical Analysis

The statistical software package SPSS 20.0.0 for Macintosh (SPSS Inc., Chicago, IL, United States) was used for all of the statistical analyses. The data are expressed as the mean and the standard error of the mean (SEM). The normality of the data was analyzed by using the Kolmogorov-Smirnov test. Comparisons between timepoints and between sexes were performed using the Student’s t-test or Mann-Whitney *U* test for the continuous variables. Significance was accepted with *p* < 0.05.

## 3 Results

These results are divided into two main groups, depending on whether they are related to the identification of equipment properties or to the effects produced at the cellular level.

### 3.1 Nanoparticle Determination

**FIGURE 1:**
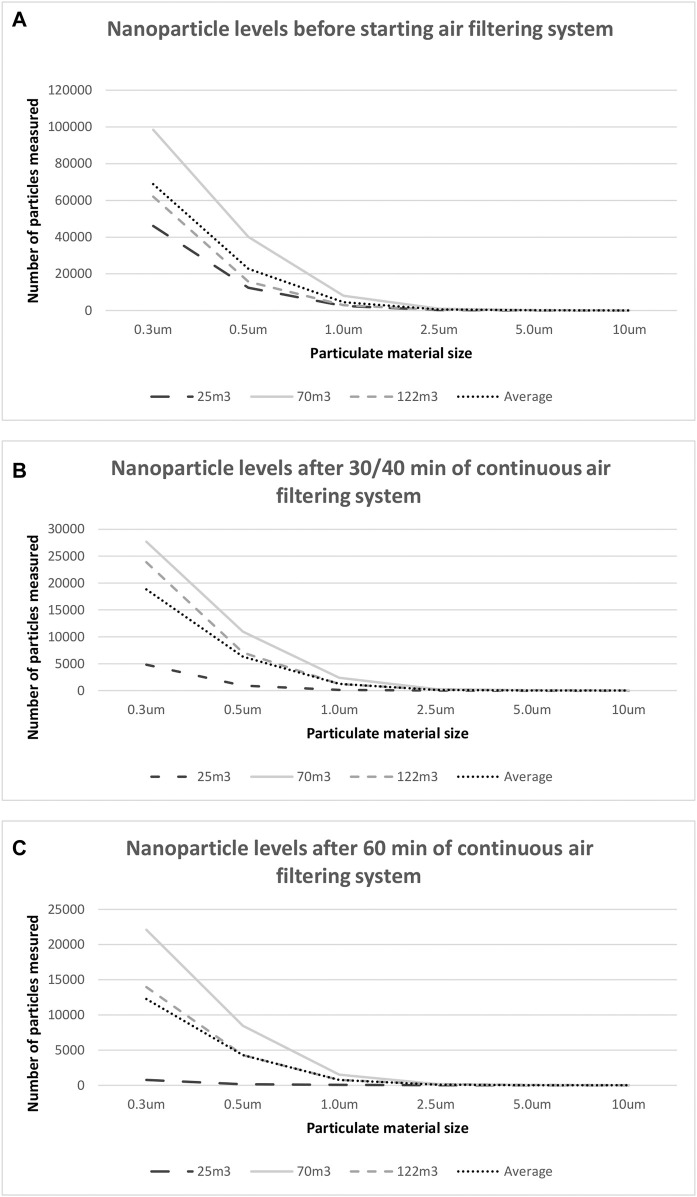
Nanoparticle levels before starting air filtering system and after 30/40/60 min of continuous use air filtering system. Results from the detection test for particulate matter (PM) with different sizes (0.3, 0.5, 1.0, 2.5, 5.0, and 10 um) in the air present in several indoor 215 spaces; 25 m^3^ (dotted thick), 70 m^3^ (dashed thick), 122 m^3^ (dotted thin) and the average value (dashed thin). Total number of particles was measured at a rate of 2.83 L per minute and every 217 21 s, with intervals of 0.99 L. **(A)** before air filtering system; **(B)** after 30/40 min and 218 **(C)** after 60 min of continuous air filtering system.

Before starting the study, nanoparticle detection tests were performed. The tests that were performed on environmental samples for PM in suspension showed a fast and effective reduction for PM of sizes 0.3, 0.5, 1.0, 2.5, 5.0 and 10 µm in indoor spaces after 30, 40 and 60 min, being PM referring to particles in suspension with a diameter smaller than the number that accompanies it. Figure 1 shows the levels of the different sizes of PM before the initiation of the system (Figure 1A), after 30/40 min (Figure 1B) and after 60 min (Figure 1C). The controls and tests showed that the volumetric flow rate, the air velocity and the outlet temperature of the system were 156.8, 224.5, and 284, 7 m^3^/h for an average flow rate during the operation of the system at speeds 1, 2 and 3, respectively, and a temperature of 24.8°C ± 0.5°C. The results of this study showed very low levels of indoor airborne nanoparticles; in fact, the obtained levels of nanoparticles were maintained below the minimum limit established by the WHO for indoor spaces. Furthermore, a higher PM load in suspension indicated a greater reduction percentage that was obtained with the system.

### 3.2 Blood Studies

#### 3.2.1 Oxidative Stress

Ionizing gas with high electrical energy based on the nature of the electrons, ions and neutral species modifies any pathway that is directly or indirectly controlled by or related to reactive oxygen species (ROS) (Vandamme et al., 2012). Therefore, the study of the variations in the level of oxidative stress induced at the systemic level should be the first step in the investigation.

**FIGURE 2:**
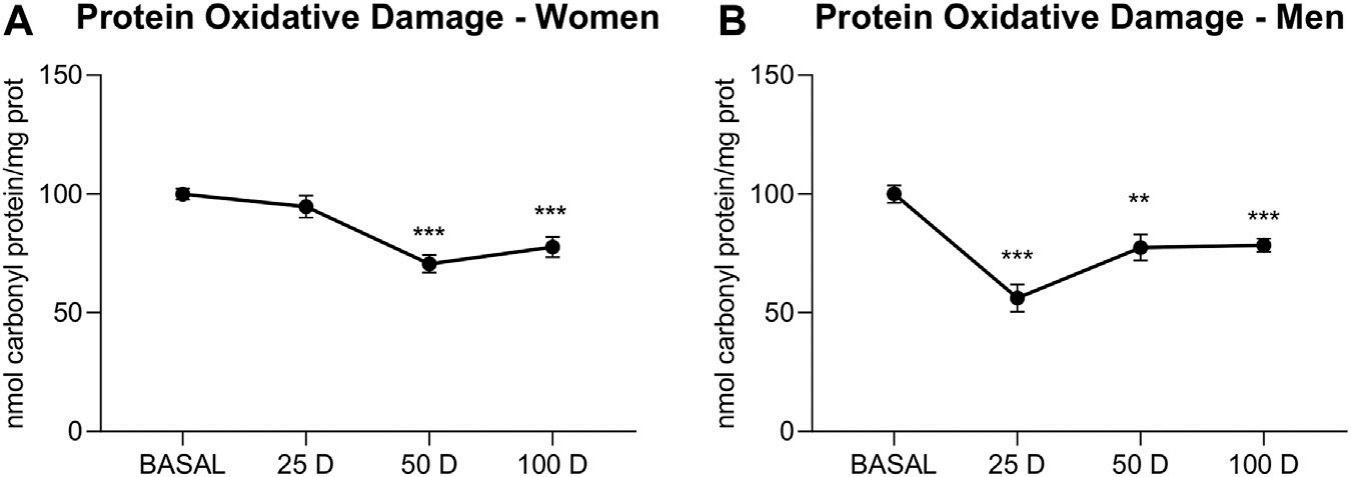
Protein oxidative damage (expressed as nmol carbonyl protein/mg prot) of plasma extracted from women **(A)** and men **(B)** at baseline (BASAL), 25 days (25 D), 50 days (50 D) and 100 days (100 D). Data are mean ± SEM. *BASAL vs. 25, 50 and 100 D. The number of symbols marks the level of significance: one for *p* < 0.001.

The results showed a significant decrease in PD (Figure 2) in all of the BioW groups; however, there were temporal differences regarding gender, due to the fact that men showed a significant reduction as early as 25 days (25 days *p* < 0.001; 50 days *p* < 0.01; 100 days *p* < 0.01), whereas in women, it was delayed until 50 days (50 days *p* < 0.01; 100 days *p* < 0.01).

**FIGURE 3:**
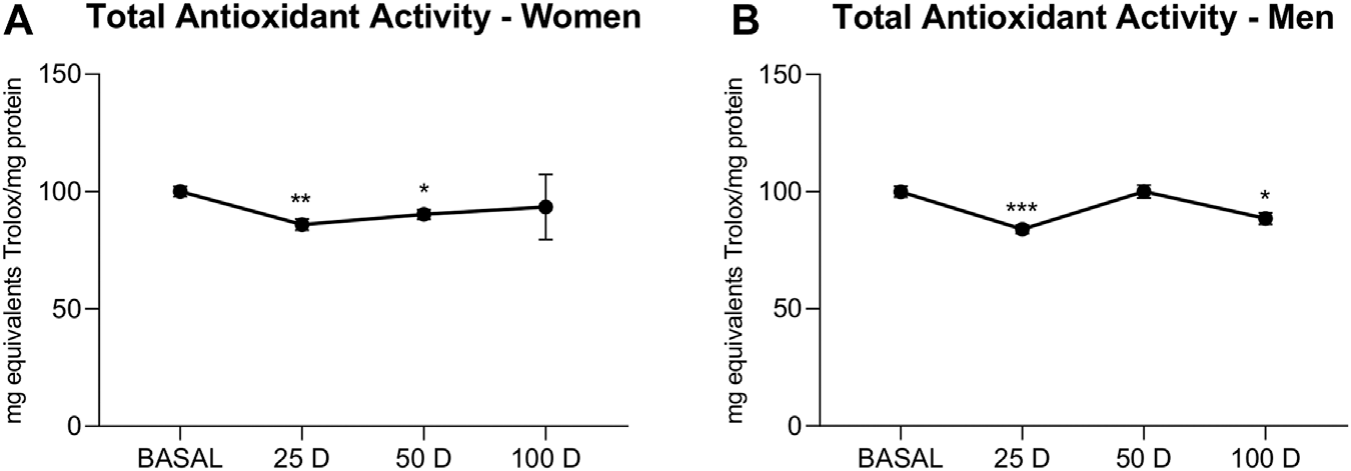
TAA (expressed as mg Trolox (Tx)/g prot) of plasma extracted from women **(A)** and men **(B)** at baseline (BASAL), 25 days (25 D), 50 days (50 D) and 100 days (100 D). Data are mean±SEM. *BASAL vs. 25, 50 and 100 D. The number of symbols marks the level of significance: one for *p* < 0.05, two for *p* < 0.01 and three for *p* < 0.001.

Total antioxidant activity showed a generalized downward trend in the BioW groups (Figure 3), which was only significant at 25 and 50 days, in women (25 days *p* < 0.01; 50 days *p* < 0.05) and at 25 (*p* < 0.001) and 100 (*p* < 0.05) in men.

### 3.2.2 Protein Synthesis

**FIGURE 4:**
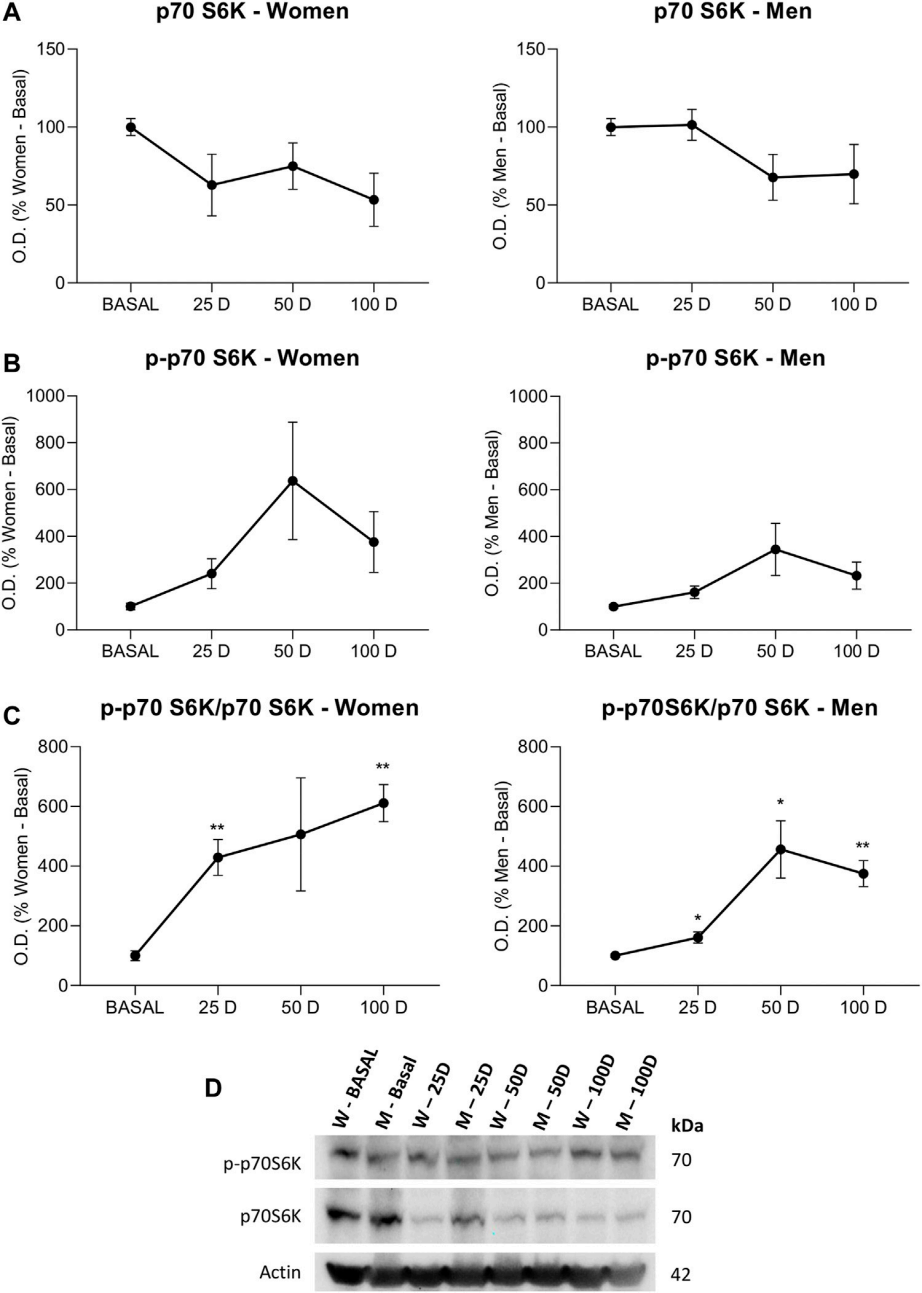
Western blot analysis for studying protein synthesis pathway in lymphocytes extracted from women and men at baseline (BASAL), 25 days (25 D), 50 days (50 D) and 100 days (100 D). Bar chart showing the optical density (OD) (Arbitrary units) of blot bands of **(A)** p70S6K, **(B)** p—p70S6K and **(C)** p—p70S6K/p70S6K, **(D)** Images of representative inmunoblots. Actin was used as a loading control. Data are mean ± SEM. *BASAL vs. 25 D, 50 D and 100 D. The number of symbols marks the level of significance: one for *p* < 0.05, two for *p* < 0.01 and three for *p* < 0.001.

p70S6K is activated by phosphorylation at threonine 389 (p-p70S6K), which induces protein synthesis and is commonly used as a marker of mTOR activation. However, this activation is directly dependent on the amount of total p70S6K in the medium; thus, the p-p70S6K/p70S6K ratio is the real indicator of the activation of protein synthesis (Figure 4).

The expression levels of p70S6K showed a clear decrease in the BIOW-treated groups, which was progressive in males and abrupt from 25 days in females (Figure 4A). The phosphorylation of p70S6K showed a similar pattern in both sexes, being more marked in women but without reaching significant differences between the different time periods. (Figure 4B). The ratio showed a gradual increase over time in females that was equivalent in males, although in this gender the increase started with a certain time delay (Figure 4C). Thus, the comparison between the sexes showed that in females, the increases were abrupt, with values much higher than those observed in males.

#### 3.2.3 Mitochondrial Oxidative Phosphorylation

**FIGURE 5:**
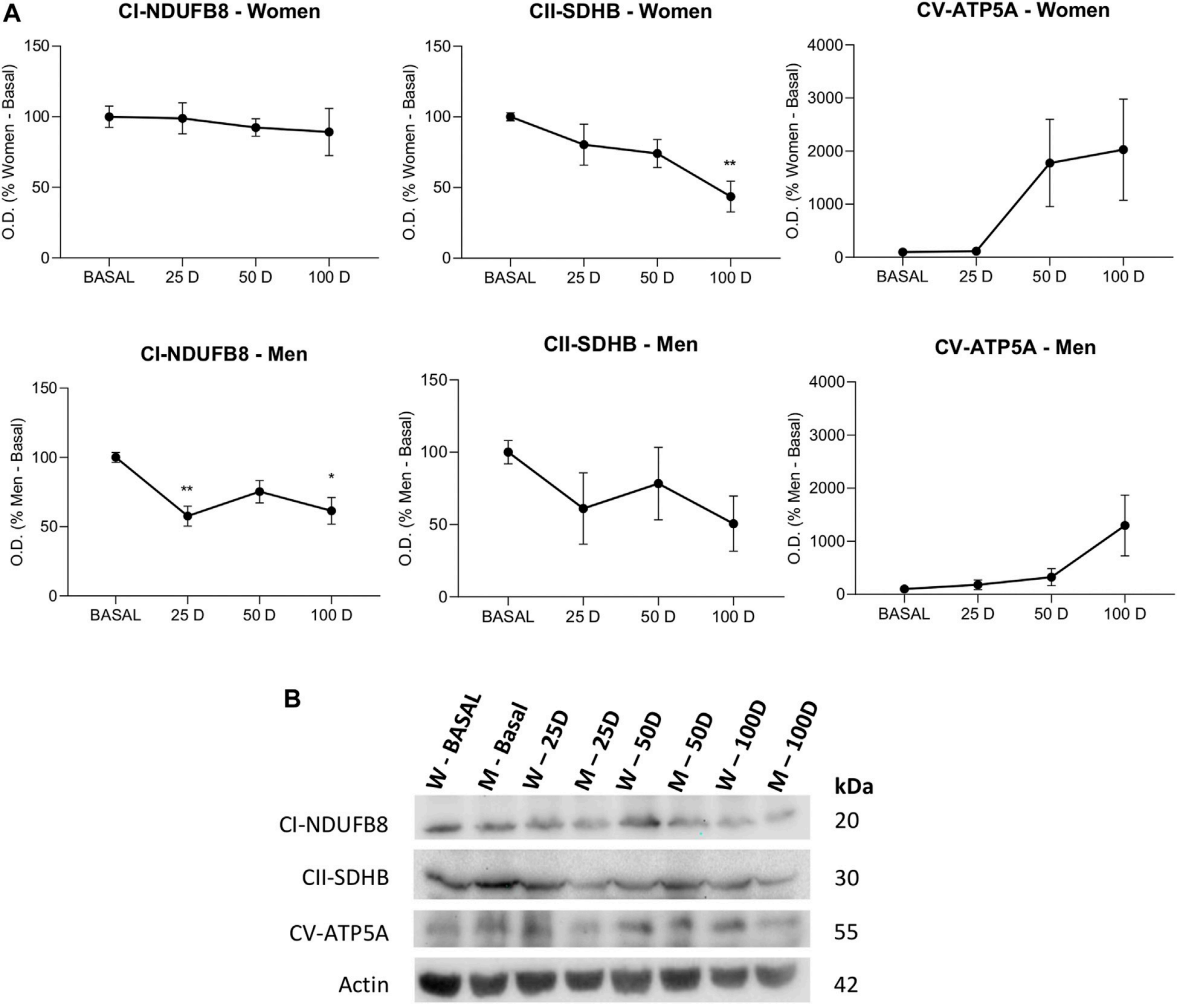
Western blot analysis for studying the protein levels of subunits from complexes of the mitochondrial electron transport chain in lymphocytes extracted from women and men at baseline (BASAL), 25 days (25 D), 50 days (50 D) and 100 days (100 D). Bar chart showing the optical density (OD) (Arbitrary units) of blot bands of **(A)** (NADH dehydrogenase ubiquitone) 1b subcomplex 8 (NDUFB8) from complex I (CI), iron sulfur subunit (SDHB) from complex II (CII) and ATP synthase subunit α (ATP5A) from complex V (CV). **(B)** Images of representative inmunoblots. Actin was used as a loading control. Data are mean ± SEM. *BASAL vs.25, 50 and 100 D. The number of symbols marks the level of significance: one for *p* < 0.05 and two for *p* < 0.01.

We hypothesized that the mitochondria could increase their efficiency with the reduction of environmental toxic elements; thus, the expression of subunits of the mitochondrial electron transport chain complexes was evaluated. Immunoblotting revealed that mitochondrial complexes I, II and V varied slightly in activity over time, showing a similar pattern in both sexes. While complex I and II showed small reductions, being significant this reduction in complex I for men (25 days *p* < 0.001; 100 days *p* < 0.05) and in complex II for women (100 days *p* < 0.01), complex V showed an increase in its activity, mainly after 50 days in both sexes without reaching significant levels (Figure 5).

### 3.2.4 Energy Production

**FIGURE 6:**
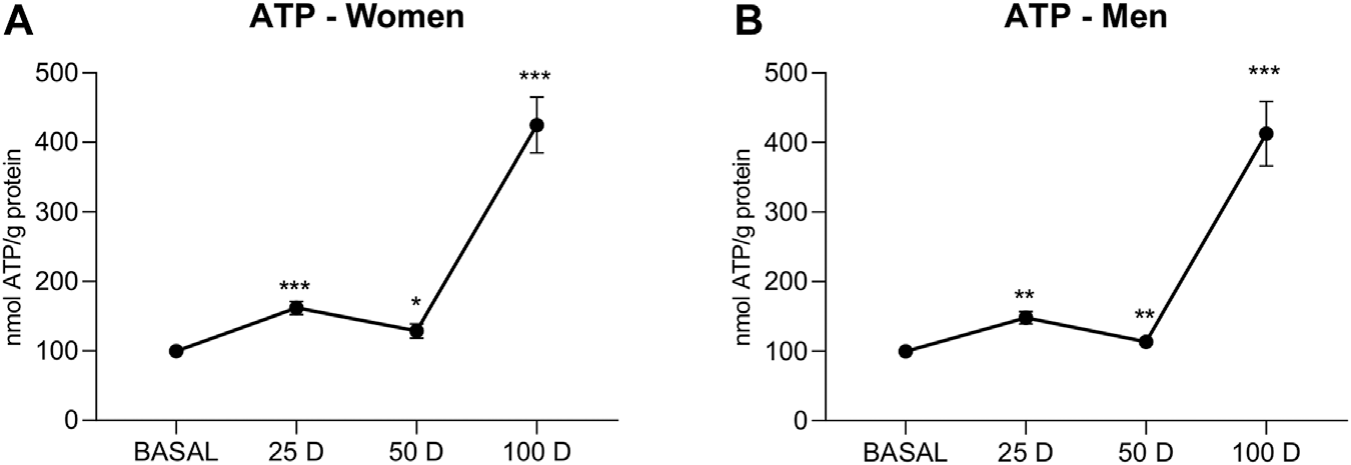
ATP content (expressed as nmol ATP/g protein) of lymphocytes extracted from women **(A)** and men **(B)** at baseline (BASAL), 25 days (25 D), 50 days (50 D) and 100 days (100 D). Data are mean ± SEM. *BASAL vs.25, 50 and 100 D. The number of symbols marks the level of significance: one for *p* < 0.05, two for *p* < 0.01 and three for *p* < 0.001.

To characterize energy levels, ATP production was studied (Figure 6). The increase in ATP levels showed a similar pattern for both sexes, with a significant increase compared to basal levels at 25 days of treatment (*p* < 0.001 for women; *p* < 0.01 for men), a decreased again at 50 days (*p* < 0.05 for women; *p* < 0.01 for men), and an abrupt spike at 100 days of treatment with BioW (*p* < 0.001 in both sexes), representing an increase of fourfold in men and women compared with the baseline levels.

## 4 Discussion

The extraordinary increase in pollutants with which human beings must coexist on a daily basis (especially in industrialized areas), encompassed under the term pollution, presents a toxic environment that has a multifactorial effect on health (Hoet et al., 2004). This level of toxicity has recently been compounded by alterations in the physical and chemical properties of apparently innocuous products, thus leading to an increase in toxicity at the nanoscopic level that can elicit harmful consequences for workers, the environment and society in general (Service, 2004; Lee et al., 2005). This environmental contamination favors the development of gastrointestinal diseases (Bhavsar and Amiji, 2007), dermatitis (Monteiro-Riviere, 2021) and eye disorders (Järvinen et al., 1995), which can significantly reduce the immune capacity against infections (Favarato et al., 2021; Katoto et al., 2021) and enhance the development of tumor processes (Coleman et al., 2021; Khorrami et al., 2021; Rojas-Rueda et al., 2021). Recent results have highlighted the important toxic role played by ultrafine particles and nanoparticles in these air pollution-induced pathological processes (Maher et al., 2020).

The use of nanofiltration equipment is very effective in reducing the pathological processes related to environmental pollution, such as silicosis or viral infections (Boczkowski and Hoet, 2010; Fermo et al., 2021), whereas exposure to nanoparticles has been shown to be a major risk for morbidity and mortality (Nishihama et al., 2021). However, the beneficial effects of the removal of such nanoparticles for healthy subjects or in the absence of pathologies are not currently known.

Studies showing a direct relationship between exposure to air pollution and an increase in ROS (Møller et al., 2010) are abundant. These highly unstable species can damage all types of biological macromolecules in their vicinity, thus causing a high degree of cellular deterioration (Hardeland et al., 2003). In humans, biomonitoring studies have demonstrated a clear association between wood smoke particles or air pollution and oxidative damage at the deoxynucleotide and lipid levels, which significantly reduces both the quality of life and the future health prospects of the population subjected to these effects. Different nanoparticles have shown an oxidative effect on biological membranes by increasing the protein carbonylation induced by oxidative damage to these molecules; these effects can, in some cases, be reversed with antioxidants (Kamat et al., 2000). Likewise, *in vitro* studies have shown that, among the effects of nanoparticles, a reduction in the antioxidant capacity can increase the initial damage to proteins (Møller et al., 2010). However, our results showed a significant and direct time-dependent reduction in oxidative damage to proteins in both male and female BioW subjects. Curiously, the decline in protein oxidative damage found in women begins at 50 days, exhibiting a delay compared to men. The described delay may be due to the different gender-based susceptibility to stressors, in which sex hormones seem to play an critical role (Institute of Medicine (US) Committee on Gender Differences in Susceptibility to Environmental Factors, 1998; Kingston et al., 2017).

Protein synthesis, which is mainly performed by the endoplasmic reticulum, is a delicate process that is easily uncoupled and hampered. From viral or bacterial infections (Chamberlain and Anathy, 2020; Alshareef et al., 2021) to the development of any neurodegenerative disease (Ghemrawi and Khair, 2020; Schneider et al., 2021), reticulum stress is a common cellular manifestation of such pathologies. Endoplasmic reticulum stress regulates mTOR, which in turn, modulates cell growth and protein synthesis through the phosphorylation of p70S6K. Moreover, mTOR/p70S6K signaling is tightly involved in the regulation of cellular quality control processes. The inhibition of mTOR and the reduction in energy production triggers autophagy that acts as a survival mechanism and ultimately decides whether apoptosis should proceed or not (Duan et al., 2016; Prieto-Domínguez et al., 2017). The recovery of the control of the protein synthesis is much more complicated and has been demonstrated in few cases, such as with treatment with melatonin, which is a potent antioxidant whose activity as a recovery factor of endoplasmic activity as an autophagy regulator has been widely demonstrated (Aouichat et al., 2021). However, this increase requires energy in all cases.

Energy production, whether aerobic or anaerobic, is essential for cell survival, and no pathologies associated with energy overproduction have been described. The finding of increased ATP levels with temporary exposure to BioW can only be related to enhanced mitochondrial functioning and efficient metabolic activity upon the removal of environmental nanoparticles (Møller et al., 2010). The reduction in ATP synthesis due to mitochondrial damage in a nanoparticle-rich environment has been known for a long time (Ueng et al., 1997). Thus, nanoparticles can induce a reduction in the efficiency of the electron transport chain that is accompanied by an inhibition of the ADP transporter into the mitochondria, thus leading to an increase in the production of oxidative stress (Hussain et al., 2005) that can trigger cell death via apoptosis (Xia et al., 2004).

It should be emphasized that in our study, there were no significant variations in the expression of OXPHOS even though its activity was necessarily increased, indicating an increase in its effectiveness. This essential difference directly correlates with ATP production, which is significantly increased with the reduction of environmental nanoparticles. Hence, the observed reduction in the expression of these complexes is evidence of improved mitochondrial efficiency. Many articles have shown a decrease in mitochondrial efficiency under stressful conditions, such as aging (Gonzalez-Freire et al., 2018) and disease.

Several agents are known to increase ATP production, including drugs that allow for recovery from disease and treatments that induce an overproduction of energy in healthy individuals. Thus, physical exercise (Vigh-Larsen et al., 2021) and various antioxidants, such as melatonin and vitamin D (Latham et al., 2021; Reiter et al., 2021), have been shown to play a dynamizing role in mitochondria by increasing ATP production in these organelles. To our knowledge, no nanofiltration equipment, except for the one used in the present study, has been shown to enhance cellular energy production. Again, the results seem to mimic an antioxidant effect at the cellular level and allow us to conclude that the effective removal of nanoparticles leads to an improvement in cellular effectiveness and efficiency, even in healthy individuals.

## 5 Conclusion

Our results have shown, for the first time to our knowledge, that the fall of environmental nanoparticles, induced by BioW remover, causes in healthy people a reduction of oxidative stress, denoted by a reduction of oxidative damage to proteins and a clear decrease in antioxidant capacity in both men and women and assessing a wide age range. In view of this oxidative environment reduction, protein synthesis and mitochondrial capacity for energy production are enhanced in a time-dependent manner. The optimization of the activity of the most oxidative stress-susceptible organelles within the cell seems to be behind both effects, suggesting an important role of environmental nanoparticles in the progressive and premature cellular depletion.

## Data Availability

The original contributions presented in the study are included in the article/Supplementary Material, further inquiries can be directed to the corresponding authors.
